# Sodium channel Na_V_1.3 is important for enterochromaffin cell excitability and serotonin release

**DOI:** 10.1038/s41598-017-15834-3

**Published:** 2017-11-15

**Authors:** Peter R. Strege, Kaitlyn Knutson, Samuel J. Eggers, Joyce H. Li, Fan Wang, David Linden, Joseph H. Szurszewski, Lorin Milescu, Andrew B. Leiter, Gianrico Farrugia, Arthur Beyder

**Affiliations:** 1 Enteric Neuroscience Program, Division of Gastroenterology & Hepatology, Department of Physiology & Biomedical Engineering, Mayo Clinic, Rochester, Minnesota United States; 2 Division of Gastroenterology, Department of Medicine, University of Massachusetts Medical School, Worcester, Massachusetts United States; 3 Department of Gastroenterology, Shanghai Tenth People’s Hospital, Tongji University School of Medicine, 300 Yanchang Middle Road, Shanghai, 200072 P.R. China; 4 Division of Biological Sciences, University of Missouri, Columbia, Missouri United States

## Abstract

In the gastrointestinal (GI) epithelium, enterochromaffin (EC) cells are enteroendocrine cells responsible for producing >90% of the body’s serotonin (5-hydroxytryptamine, 5-HT). However, the molecular mechanisms of EC cell function are poorly understood. Here, we found that EC cells in mouse primary cultures fired spontaneous bursts of action potentials. We examined the repertoire of voltage-gated sodium channels (Na_V_) in fluorescence-sorted mouse EC cells and found that *Scn3a* was highly expressed. *Scn3a*-encoded Na_V_1.3 was specifically and densely expressed at the basal side of both human and mouse EC cells. Using electrophysiology, we found that EC cells expressed robust Na_V_1.3 currents, as determined by their biophysical and pharmacologic properties. Na_V_1.3 was not only critical for generating action potentials in EC cells, but it was also important for regulating 5-HT release by these cells. Therefore, EC cells use *Scn3a*-encoded voltage-gated sodium channel Na_V_1.3 for electrical excitability and 5-HT release. Na_V_1.3-dependent electrical excitability and its contribution to 5-HT release is a novel mechanism of EC cell function.

## Introduction

More than 90% of the serotonin (5-hydroxytryptamine, 5-HT) in the body is produced and released into circulation by a single enteroendocrine cell type in the gastrointestinal (GI) tract—the enterochromaffin (EC) cell^1^. EC cell 5-HT plays critical roles in a variety of physiologic processes^2^. In the gut, EC cell 5-HT is a paracrine and neurotransmitter molecule that is involved in several GI functions, such as motility, secretion and sensation^3–6^. EC cell 5-HT is also an important hormone in a variety of organ systems, regulating cardiovascular tissue function^7^, bone health^8^, and metabolism^9,10^. Targeting EC cell 5-HT is emerging as a novel and promising therapeutic option for a diverse set of pathologic conditions, ranging from mucosal inflammation^11^ to osteoporosis^8^ and metabolic dysfunction^9^. However, a deep molecular understanding of EC cell function is currently lacking.

Similar to other endocrine cells, EC cells release 5-HT by two forms of exocytosis: Ca^2+^-dependent and Ca^2+^-independent^12^. The Ca^2+^-dependent form of exocytosis relies on activation of voltage-gated calcium channels^13,14^ and accounts for most of the released 5-HT^15^. However, the molecular mechanisms upstream of calcium channel activation are unclear. Previous studies showed that other endocrine^16^, neuroendocrine^17^, and enteroendocrine cells^18^ are electrically excitable, with voltage-gated sodium (Na^+^) channels contributing to electrical excitability and stimulation-secretion coupling^17^.

The goal of this study was to determine whether EC cells are electrically excitable and, if so, whether voltage-gated sodium channels (Na_V_) were responsible for EC cell excitability. We present several lines of evidence that a single voltage gated sodium channel, the *Scn3a*-encoded Na_V_1.3, is highly and specifically enriched in EC cells, renders the EC cells electrically excitable, and contributes to 5-HT release. Parts of this study were previously presented^19–21^.

## Results

### Murine enterochromaffin (EC) cells show spontaneous electrical excitability

**Figure 1:**
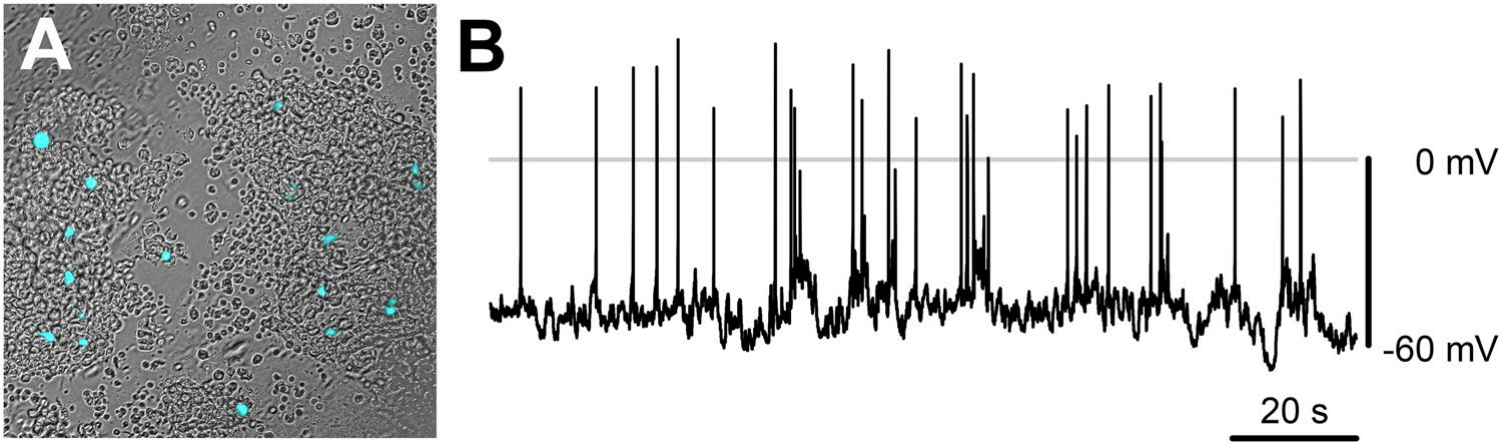
Primary EC cells have spontaneous electrical excitability. (**A**) Overlaid DIC/epifluorescence image of a primary colon culture from a Tph1-CFP mouse. CFP fluorescent cells (*cyan*) are EC cells. (**B**) Spontaneous electrical activity from a murine primary small bowel EC cell (whole cell current clamp).

To examine EC cell function, we developed murine small intestine primary cultures from a Tph1-CFP mouse transgenic model^22^. In this model, all cells that express tryptophan hydroxylase 1 (*Tph*1), which is specific to peripheral serotonin-producing EC cells^1^, also express CFP. We could easily identify CFP+ EC cells in primary cultures (Fig. 1A). We first examined EC cell electrical excitability using current-clamp. Given the epithelial lineage of EC cells, we were surprised to find that EC cells frequently showed spontaneous bursts of fast action potentials (Fig. 1B). In view of these results, we pursued experiments to investigate the molecular nature of EC cell excitability.

### Murine EC cells express the sodium channel gene *Scn3a*

**Figure 2:**
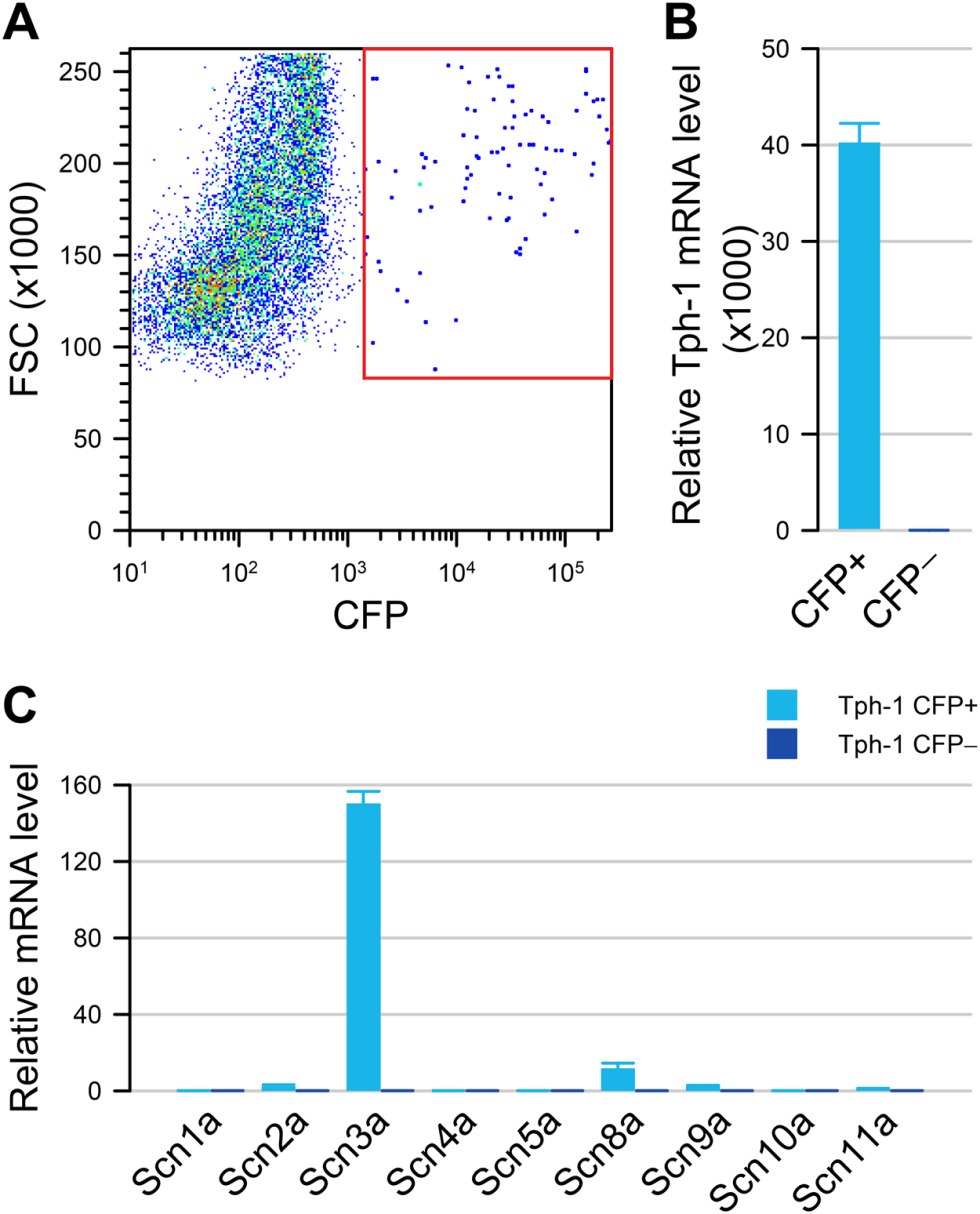
Tph1-CFP EC cells highly express *Scn3a*. (**A**) Fluorescence-activated sorting of dissociated small bowel epithelial cells from Tph1-CFP mouse showing that 0.74% are CFP+ cells. (**B**) In the CFP+ fraction (*cyan*), Tph1 is highly expressed compared to CFP− (*blue*) cells. (**C**) Voltage-gated sodium channel α-subunit mRNA levels in CFP+ (*cyan*) and CFP− (*blue*) cells, showing a high expression of *Scn3a* in CFP+ EC cells but not in CFP− cells.

We next aimed to find out whether EC cell electrical excitability was due to expression of voltage-gated sodium channel α-subunits. We dissociated Tph1-CFP small bowel and isolated CFP+ cells using fluorescence activated sorting. We found that 0.74 ± 0.23% (N = 6) of all sorted cells were CFP+ (Fig. 2A). In the CFP+ cells, *Tph1* mRNA was highly enriched (20,249 ± 2,018-fold, N = 3) compared to CFP- cells confirming that CFP+ cells are highly enriched EC cells (Fig. 2B). We found that the CFP+ EC cells highly expressed *Scn3a* mRNA, a gene encoding voltage-gated sodium channel Na_V_1.3, (1,593.4 ± 51.9-fold, N = 3) compared to CFP- cells. The most highly expressed gene among the family of all sodium channel α-subunits was *Scn3a*, expressed 150.3 ± 6.2-fold higher (N = 3) than other sodium channel genes (e.g. *Scn11a*), which were found at trivial levels in CFP+ cells (Fig. 2C). No significant expression of any voltage-gated sodium channel α-subunit mRNA was found in the CFP- cells (Fig. 2C). Our data, therefore, show that *Scn3a* is abundantly and specifically expressed in EC cells.

### Human and mouse colon and small bowel EC cells express voltage-gated sodium channels (Na_V_)

**Figure 3:**
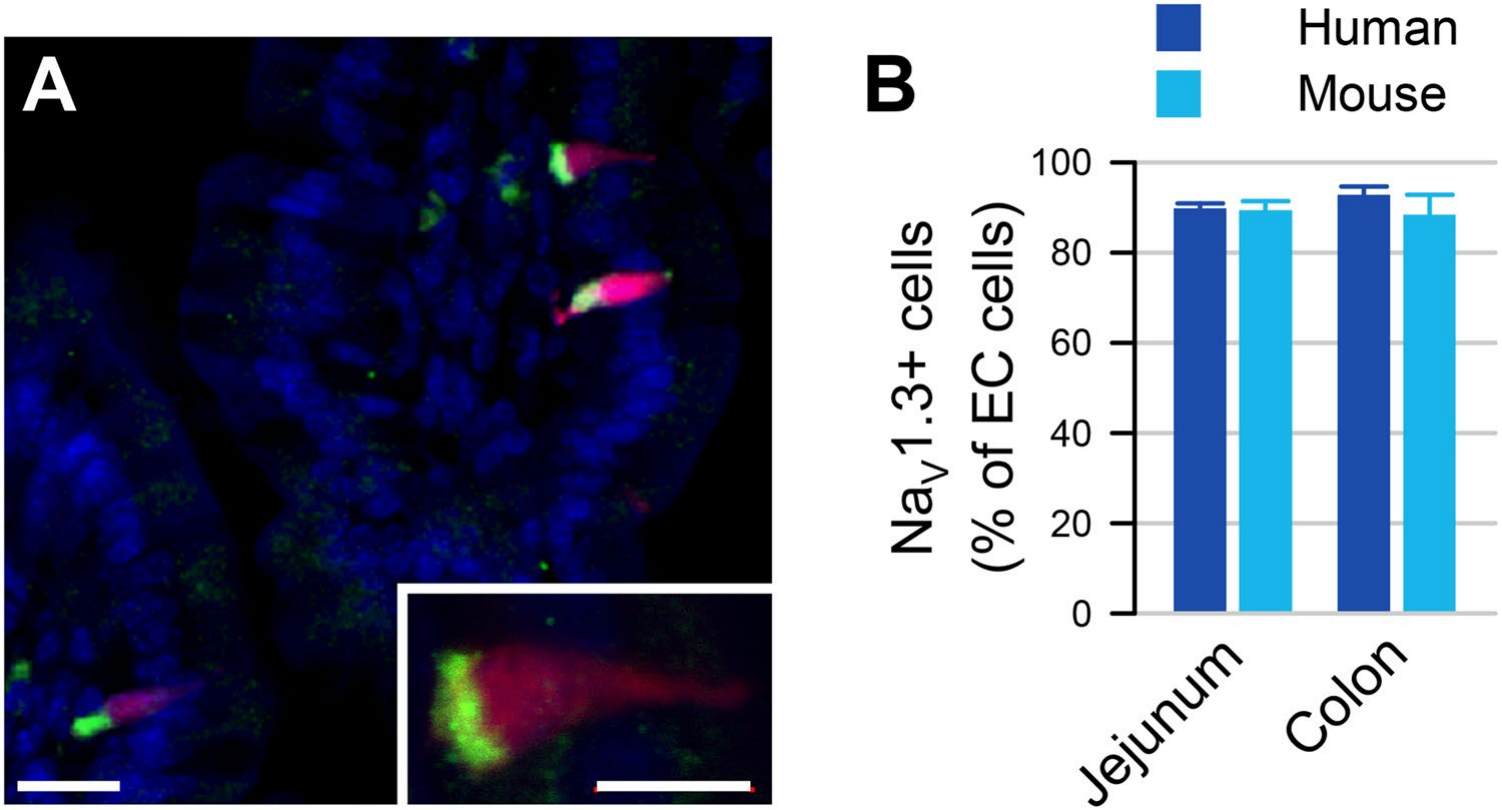
*Scn3a*-encoded Na_V_1.3 is specific to EC cells of mouse and human. (**A**) Expression of EC cell marker Tph-1 and voltage-gated sodium channel gene *Scn3a* by RNAseq in FACS-sorted Tph1-CFP EC cells from mouse small bowel. *Red*, TPH1-CFP; *green*, Na_V_1.3; *blue*, DAPI. *Scale bar*, 10 µm; *inset*, 5 µm. *Inset*, Na_V_1.3 is localized at the basal side of mouse EC cells. (**B**) Percentages of Na_V_1.3+/5HT+ cells out of all EC (5HT+) cells in human (*blue*) and Na_V_1.3+/CFP+ cells out of all EC cells (CFP+) in mouse (*cyan*) jejunum (*left*) and colon (*right*), as counted from immunohistochemistry data.

We used immunofluorescence to determine whether Na_V_1.3 protein is present in EC cells of human and mouse colon and small bowel (Fig. 3A). We found that Na_V_1.3 is not only present in both mouse and human, but it appears to be localized highly asymmetrically — almost exclusively at the basal side (Fig. 3A). In the mouse and human GI epithelium, we found that Na_V_1.3 was present in most EC cells (mouse Tph1-CFP+ and human 5-HT+ cells) in both small bowel and colon (Fig. 3B). We quantified the frequency of CFP+/Na_V_1.3+ cells and found co-localization in 89.4 ± 2.0% of small bowel EC cells (N = 3 animals, n = 71 ± 5 cells/animal) and 88.4 ± 4.4% of colon EC cells (N = 3 animals, n = 73 ± 5 cells/animal) (Fig. 3B). Similarly, in the human GI epithelium, we found that Na_V_1.3 and 5-HT co-localized in 89.8 ± 1.1% of small bowel EC cells (N = 3 patients, n = 70 ± 3 cells/patient) and 92.8 ± 2.0% of colon EC cells (N = 3 patients, n = 68 ± 5 cells/patient) (Fig. 3B). Altogether, our data from the human and mouse small bowel and colon show that ~90% of EC cells express the voltage-gated sodium channel Na_V_1.3.

### Primary colon and small bowel EC cells express *Scn3a* and have robust Na_V_1.3 currents

**Figure 4:**
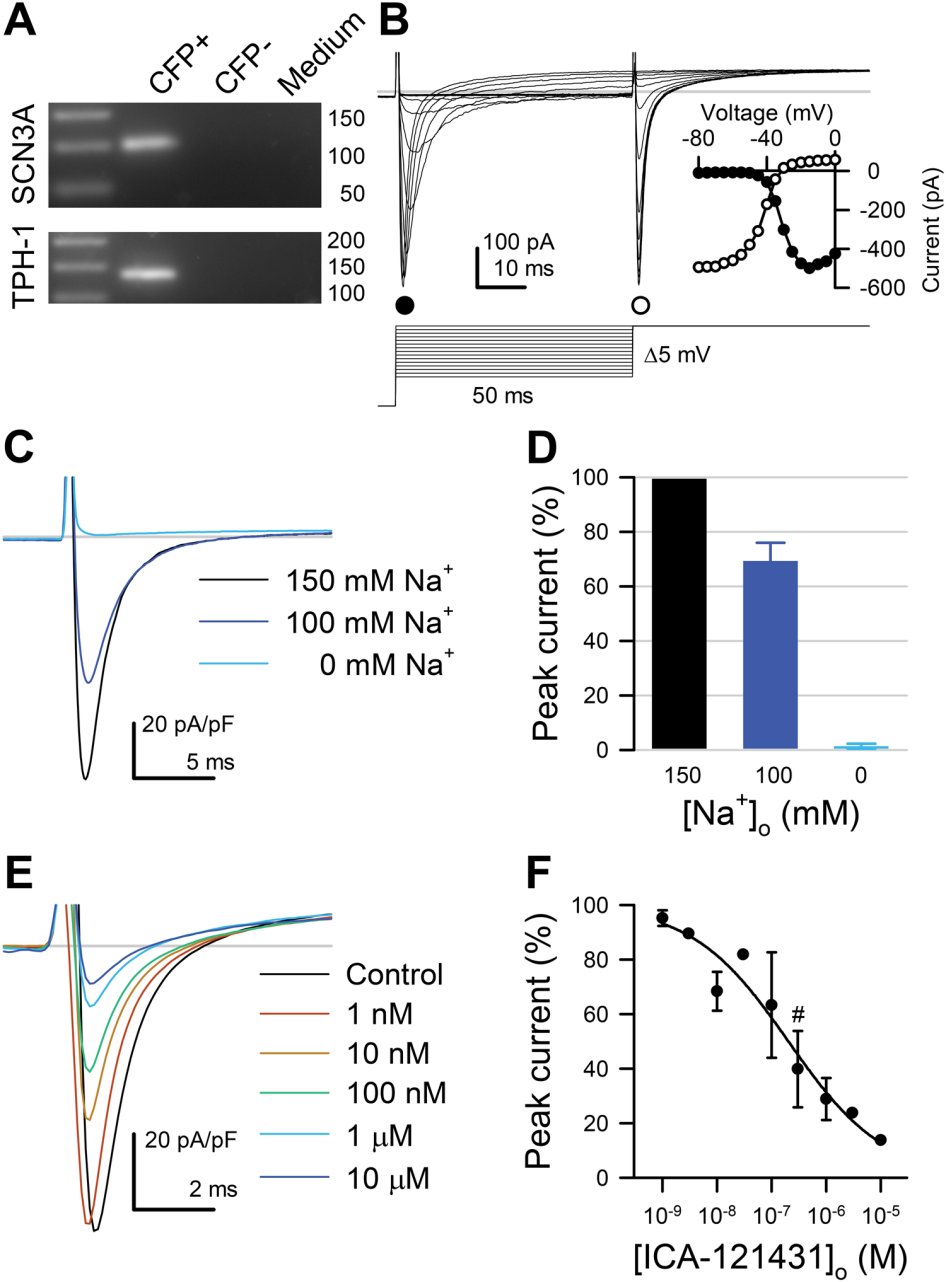
Primary cultured mouse small bowel EC cells express *Scn3a* and have fast voltage-gated inward currents that are selective for Na^+^ and inhibited by the Na_V_1.3 blocker ICA-121431. (**A**) Cropped single cell RT-PCR gel of *Tph1-CFP*+, *Tph1-CFP-* cells, or *medium*. (**B**) Representative traces of fast inward currents of voltage-clamped Tph1-CFP+ (EC) cells, elicited by a 2-step voltage ladder protocol (*bottom*). *Inset*, Peak currents of the traces shown in *B*, elicited during the activation (*step 1*, ●) or inactivation (*step 2*, ○) steps, plotted versus the voltage of *step 1*. (**C**) Representative traces of inward currents elicited by a step from −120 to −20 mV with 150 (*black*), 100 (*blue*), or 0 mM (*cyan*) extracellular [Na^+^]. Replacement of extracellular Na^+^ with *N*-methyl *D*-glucamine (NMDG^+^) diminishes (100 mM Na^+^) or eliminates (0 mM Na^+^) the fast inward currents. (**D**) Average peak Na^+^ current at the −20 mV test pulse with 100 mM [Na^+^]_o_ (*blue*) or 0 mM [Na^+^]_o_ (*cyan*), normalized to the peak Na^+^ current recorded with 150 mM [Na^+^]_o_ (*black*) (n = 4). (**E**) Representative Na^+^ currents elicited from small bowel EC cells by steps from −120 to −20 mV with 0 (*black*) or 10^−9^ to 10^−5^ mol/L Na_V_1.3 blocker ICA-121431 (*color gradient*). (**F**) Dose-response of peak Na^+^ currents to ICA-121431 (IC_50_, 131 ± 54 nM; maximum block, 68 ± 7%, n = 4). Symbol (#) denotes 0.3 µM ICA-121431, the concentration used in Fig. 5D.

To directly confirm *Scn3a* expression in EC cells, we used single cell RT-qPCR in Tph1-CFP mouse small bowel and colon primary cultures. We found that *Scn3a* and *Tph1* mRNA were present in CFP+ EC cells but not CFP- cells or bath medium from both mouse small bowel (N = 3) and colon (N = 3) primary cell cultures (Fig. 4A, full size gel in Supplementary Figure 1).

To evaluate the functional role of Na_V_1.3 in EC cell excitability, we performed whole cell voltage-clamp experiments on CFP+ EC cells from mouse small bowel and colon and compared their electrophysiologic properties (Table 1). We found fast voltage-dependent inward currents in 128 of 154 small bowel EC cells (81.3 ± 4.0%, N = 44 cultures) and 18 of 29 colon EC cells (64.1 ± 9.2%, N = 29 cultures) (Fig. 4B). CFP- cells in the same preparations did not have voltage-dependent inward currents (Supplementary Figure 2). The peak currents were −63.4 ± 5.6 pA/pF for small bowel and −68.0 ± 11.5 pA/pF for colon (n = 114 and 18, *p* > 0.05 by a nonparametric two-tailed t-test). Voltage-dependent channel activation was well fit by a Boltzmann function with the following parameters: half-activation voltage (V_1/2A_) −23.4 ± 0.9 mV and slope (δV_A_) 6.7 ± 0.2 mV for small bowel, V_1/2A_ −25.3 ± 2.2 mV and δV_A_ 6.5 ± 0.3 mV for colon EC cells (Fig. 4B inset, Table 1). The time constant of activation (τ_A_) was 0.26 ± 0.02 ms for small bowel and 0.23 ± 0.03 ms for colon EC cells. Inactivation was best fit by a two-exponential function, with τ_F_ 1.00 ± 0.04 ms and τ_S_ 11.4 ± 0.6 ms for small bowel, τ_F_ 0.68 ± 0.04 ms and τ_S_ 11.2 ± 1.1 ms for colon EC cells (*p* < 0.05, τ_F_ of colon *vs*. small bowel EC cells). The voltage-dependence of inactivation, or steady-state availability, was also well-fit by a two-state Boltzmann function. The half-inactivation voltage (V_1/2I_) and slope (δV_I_) were −48.2 ± 1.1 mV and 7.1 ± 0.1 mV for small bowel, −53.3 ± 3.8 mV and 8.9 ± 0.9 mV for colon EC cells (*p* > 0.05) (Fig. 4B, inset). Overall, we found robust voltage-gated fast inward currents in colon and small bowel EC cells. These currents had similar biophysical properties in both small bowel and colon EC cells except that colonic EC cells had smaller capacitances and had faster fast inactivation time constants (Table 1, Supplementary Figure 3).

**Table 1:** Na_V_ channels in primary EC cells have similar properties.

	ECJ	ECC
Preps (n)	44	13
Cells (n)	125	18
P(I_Na_) (%)	81.3 ± 4.0	64.1 ± 9.2
C (pF)	3.9 ± 1.0	2.1 ± 0.2*
I_PEAK_/C (pA/pF)	−63.4 ± 5.6	−68.0 ± 11.5
G_MAX_ (pA/pF)	1.57 ± 0.13	1.60 ± 0.19
V_1/2I_ (mV)	−48.2 ± 1.1	−53.3 ± 3.8
δV_I_	7.1 ± 0.1	8.9 ± 0.9
V_1/2A_ (mV)	−23.4 ± 0.9	−25.3 ± 2.2
δV_A_	6.7 ± 0.2	6.5 ± 0.3
E_REV_ (mV)	39.0 ± 2.1	31.8 ± 4.3
τ_A_ (ms)	0.26 ± 0.02	0.23 ± 0.03
τ_F_ (ms)	1.00 ± 0.04	0.68 ± 0.04*
τ_S_ (ms)	11.4 ± 0.6	11.2 ± 1.1

Parameters of Na^+^ current under control conditions in CFP^+^ enterochromaffin cell cultures from mouse small bowel (*ECJ*) or colon (*ECC*). *Preps*, preparations of EC cell primary cultures. *Cells*, patch clamped murine TPH1-CFP+ EC cells with measurable Na^+^ current. *P*(*I*_*Na*_), average proportion of EC cells with measurable Na^+^ current in any given EC preparation. *C*, cell capacitance. *I*_*PEAK*_ */C*, peak Na^+^ current normalized to cell capacitance. *G*_*MAX*_, maximum conductance. *V*_*1/2I*_, voltage of half inactivation. *δV*_*I*_, slope of voltage dependence of inactivation. *V*_*1/2A*_, voltage of half activation. *δV*_*A*_, slope of voltage dependence of activation. *E*_*REV*_, reversal potential of Na^+^ current. *τ*_*A*_, time constant of activation. *τ*_*F*_, time constant of fast inactivation. *τ*_*S*_, time constant of slow inactivation. Values shown for time constants are at 0 mV. *P < 0.05 ECC *vs*. ECJ by a non-parametric two-tailed t-test.

Although RNA expression experiments suggest Na_V_1.3 as the primary sodium channel, the fast-inactivated, voltage-dependent inward currents that we observed could be carried, in principle, by either a Na^+^ or Ca^2+^ channel. Thus, we used ion substitution to determine which type of voltage-gated channel carries the current (Na^+^ or Ca^2+^), and pharmacologic blockade to identify the specific subtype (Nav1.3 or potentially others). The fast voltage-dependent inward currents in small bowel EC cells were eliminated by substituting Na^+^ with N-methyl D-glucamine (NMDG^+^) (*I*_PEAK_: 150 mM [Na^+^]_o_, −75.0 ± 34.4 pA/pF; 100 mM [Na^+^]_o_, −51.0 ± 22.2 pA/pF; 0 mM [Na^+^]_o_, −0.3 ± 1.8 pA/pF; n = 4, *p* < 0.05, 150 mM to 0 mM [Na^+^]_o_ by a repeated measures ANOVA with Dunnett post-test) (Fig. 4C). Current-voltage (I-V) relationships showed decreases in peak currents for 100 mM Na^+^ (Fig. 4C & D) without a change to voltage-dependent parameters when fit to a two-state Boltzmann function (data not shown). Full Na^+^ replacement by 150 mM NMDG^+^ eliminated the fast-inward currents (Fig. 4C & D). We next used the selective Na_V_1.3 inhibitor ICA-121431^23^ (Fig. 4E). At the + 20 mV test pulse, ICA-121431 dose-dependently inhibited the fast-inward currents with an IC_50_ of 204 ± 54 nM and block of 86.3 ± 0.1% at 10 µM, the highest concentration tested here (n = 4) (Fig. 4E & F). Together, the ionic substitution and pharmacological blockade experiments establish that the fast voltage-dependent inward current in EC cell is carried by sodium and specifically by the *Scn3a*-encoded voltage-gated sodium channel Na_V_1.3. If other channels contribute as well, their currents were not measurable.

### Na_V_1.3 channels are responsible for elicited action potentials (APs) in EC cells

**Figure 5:**
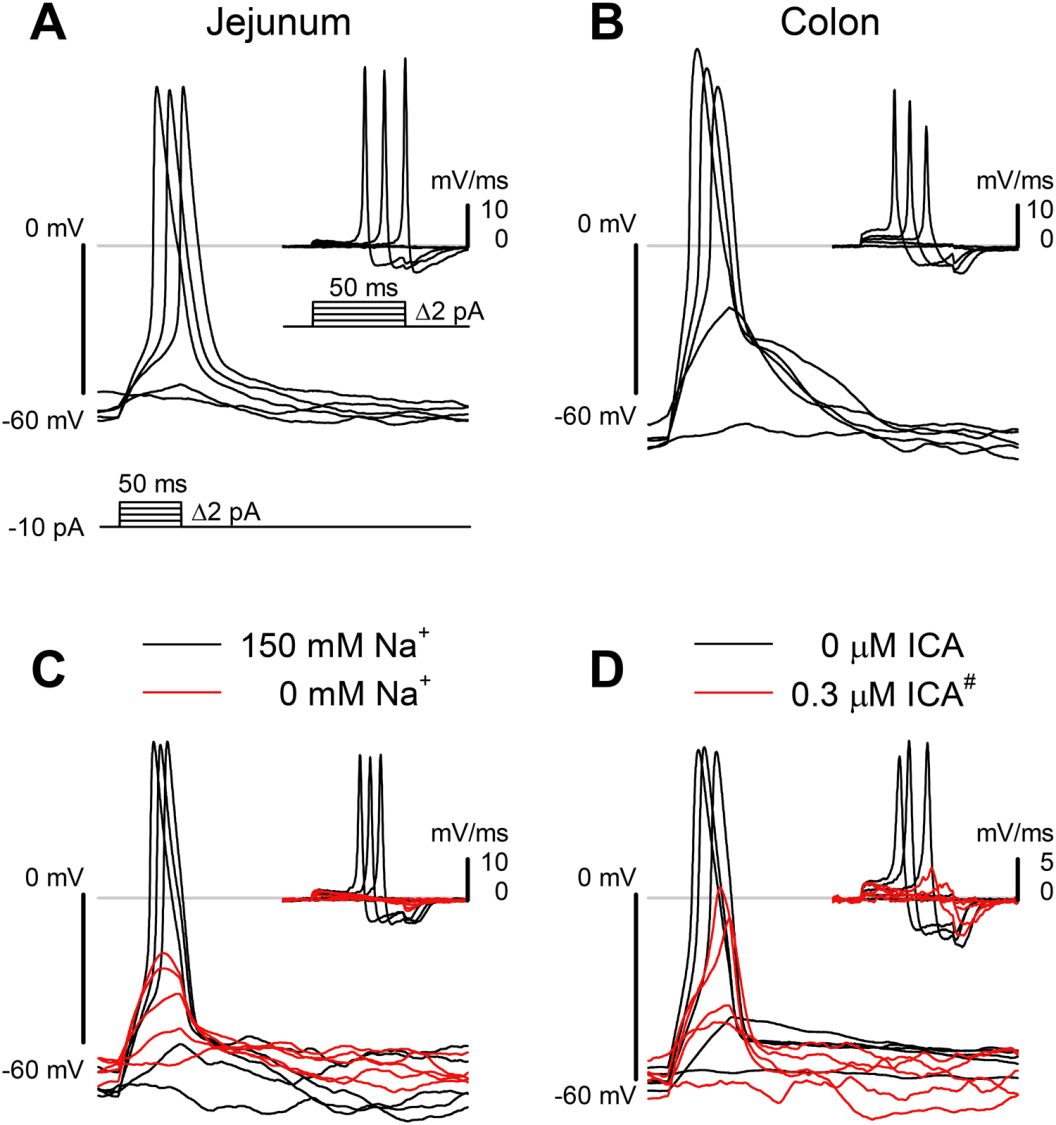
Na_V_1.3 is necessary for EC cell action potentials. (**A**,**B**) Typical action potentials from small bowel (**A**) and colon (**B**) EC cells, elicited by 50-ms depolarizing current steps (*bottom*). (**C**,**D**) Elicited EC cell action potentials were blocked by Na^+^ ion replacement (**C**) or Na_V_1.3 inhibition using ICA-121431 (300 nM, as highlighted by # in Fig. 4F). (**D**) *Insets*, first derivatives of the traces in each panel, showing the rate of change in membrane potential induced by the current injection protocol. The maximum depolarization rate is an approximate measure of the inward current through voltage-gated sodium channels.

If Na_V_1.3 is the sole source of voltage-dependent inward current in EC cells, it should then be the logical candidate for AP generation. We examined whether Na_V_1.3 channels were involved in EC cell APs. We current-clamped the EC cells from both small bowel and colon and used short pulses of current injection to elicit electrical activity. We found that for EC cells (CFP+) from both small bowel (Fig. 5A) and colon (Fig. 5B), a current stimulus of 20–50 ms resulted in action potentials in 48 of 57 cells (84.2%) in the small bowel and 5 of 7 cells (71.4%) in the colon. In the EC cells that lacked fast inward current from both colon (n = 5) and small bowel (n = 4), we were unable to elicit APs. For EC cells in which APs could be elicited, the threshold potential was not different between small intestine and colonic EC cells (ECJ, −47.8 ± 1.4 mV, n = 48; ECC, −57.7 ± 7.0 mV, n = 5; P > 0.05 t-test for unequal variance). The action potential peaks were +55.3 ± 3.1 mV and +39.1 ± 8.2 mV for small bowel and colon, respectively. To determine whether Na_V_1.3 was responsible for the EC cell action potentials, we used Na^+^ substitution (Fig. 5C) and Na_V_1.3 blockade (Fig. 5D). Both approaches resulted in action potential elimination (Fig. 5C & D).

### EC cells exhibit bursts of spontaneous electrical activity

**Figure 6:**
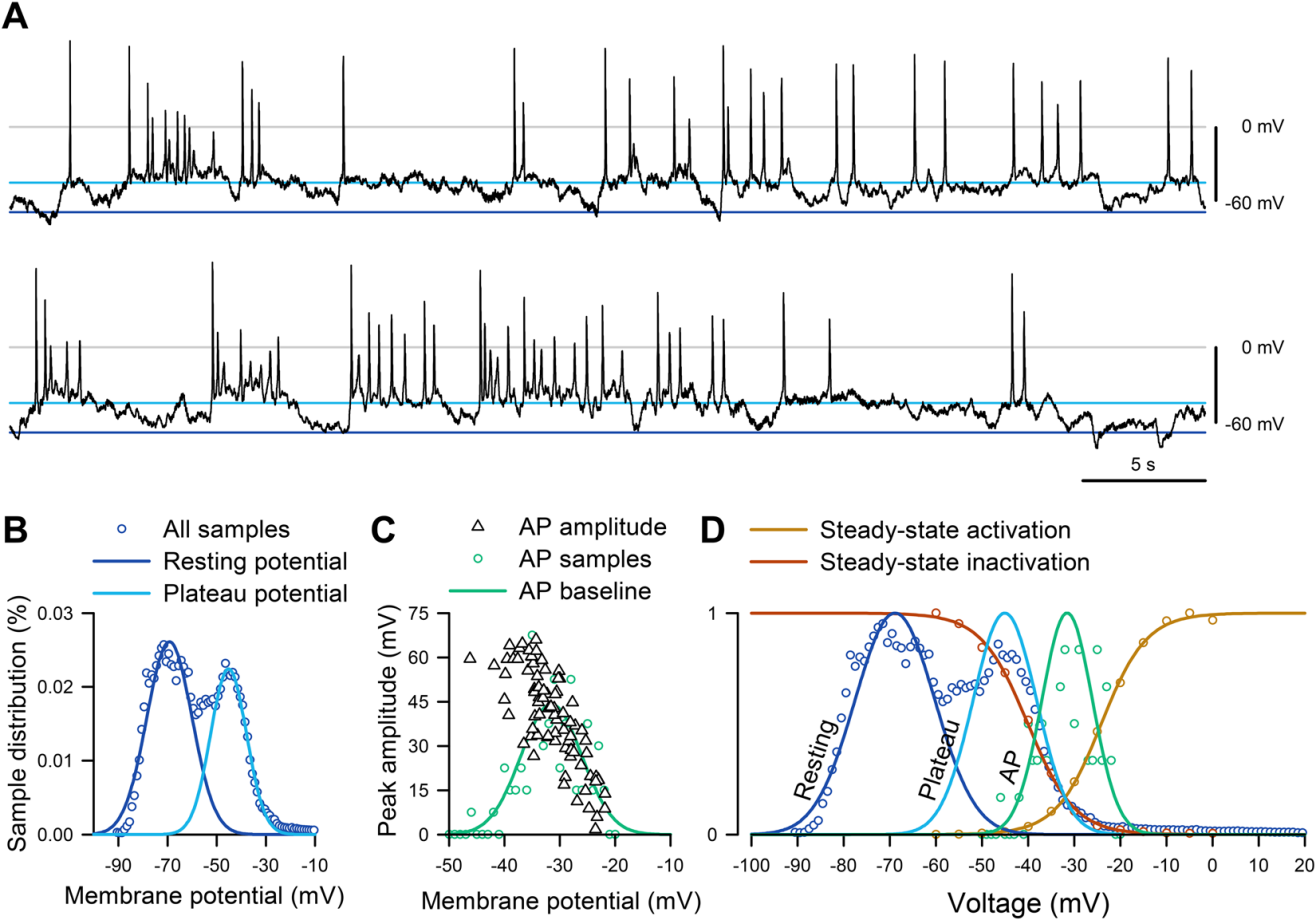
EC cells fire spontaneous action potentials. (**A**) Current clamp recording of spontaneous electrical activity in the absence of current stimulus from a Tph1-CFP^+^ EC cell from mouse small bowel. In voltage clamp mode, this EC cell had 300 pA peak Na^+^ current, −24 mV half-maximal activation (V_1/2A_), and −41 mV half-maximal inactivation (V_1/2I_) (data not shown). (**B**) Sample distribution of the membrane potential (*circles*) from the full recording of panel *A*, fit with two Gaussian functions, showing separation into resting (*blue*) and plateau (*cyan*) potentials. (**C**) Peak amplitudes of action potentials (AP, *triangles*) versus the baseline prior to the firing of each AP. Distribution of AP (*circles*, n = 0–7 per 1-mV bin) was fit with a Gaussian function (*green*). (**D**) Action potential baseline distribution overlaps Na_V_1.3 window current. Normalized steady-state activation (*red*) or inactivation (*yellow*) currents from voltage-clamp (fit with Boltzmann functions), overlaying the normalized all-sample distribution of resting (*blue*) and plateau (*cyan*) potentials from (**B**) or AP baseline (*green*) from (**C**).

Having found spontaneous electrical activity in EC cells (Fig. 1) and having established the role of Na_V_1.3 in the generation of action potentials, we examined whether Na_V_1.3 was involved in EC cell spontaneous electrical activity. Current-clamped EC cells fired spontaneous but irregular bursts of action potentials from plateau potentials (Fig. 6A), often for minutes at a time during a single experiment (Supplementary Figure 4). The EC membrane potential oscillated between a resting potential of −72 ± 4 mV and a plateau potential of −56 ± 4 mV (Fig. 6B, Supplementary Figure 5). The action potential amplitudes showed a decay along the distribution of their baselines (Fig. 6C, black). Overall, EC cells had hyperpolarized resting potentials, discrete plateau potentials, and from plateau potentials fired bursts of action potentials that had properties consistent with the voltage-dependent function of Na_V_1.3 channels examined in previous sections (Fig. 6D).

### Na_V_1.3 contributes to the regulation of 5-HT release by EC cells

**Figure 7:**
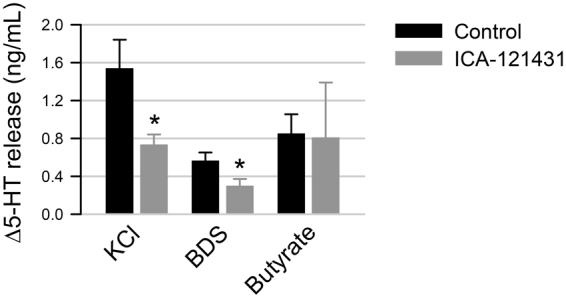
Na_V_1.3 contributes toward 5-HT release from EC cells. The change in release of 5-HT (Δ5-HT) from primary colonic EC cells was measured by ELISA following 20 min incubation with KCl (*left*),BDS-1 (*middle*) or butyrate (*right*), in the absence (*black*) or presence (*gray*) of Na_V_1.3 inhibitor ICA-121431.

To determine whether Na_V_1.3 contributes to 5-HT release, we used ELISA to measure 5-HT release from primary colon cultures (Fig. 7). Using KCl (50 mM) as a cell depolarization agent, we found that cell depolarization resulted in a large release of 5-HT from colonic EC cells (from t_0_ of 0.24 ± 0.25 ng/mL to t_20,KCl_ of 1.87 ± 0.95 ng/mL, for Δ5-HT_KCl_ of 1.54 ± 0.30 ng/mL, n = 7). This response was significantly decreased by the addition of ICA-121431 to block Na_V_1.3 (from t_0_ of 0.40 ± 0.36 ng/mL to t_20,KCl,ICA_ of 1.14 ± 0.54 ng/mL for Δ5-HT_KCl,ICA_ of 0.74 ± 0.11 ng/mL, n = 5, *p* < 0.05 between Δ5-HT_KCl_ and Δ5-HT_KCl,ICA_ by Student’s t-test). BDS-1, a known Na_V_1.3 agonist^24^, also induced 5-HT release from primary cultures (from t_0_ of 0.34 ± 0.33 ng/mL to t_20,BDS_ of 0.91 ± 0.38 ng/mL, for a Δ5-HT_BDS_ of 0.57 ± 0.08 ng/mL, n = 5). Addition of ICA-121431 significantly inhibited the response to BDS-1 (from t_0_ of 0.35 ± 0.30 ng/mL to t_20,BDS_ of 0.66 ± 0.20 ng/mL, for a Δ5-HT_BDS,ICA_ of 0.30 ± 0.07 ng/mL, n = 5, *p* < 0.05 between Δ5-HT_BDS_ and Δ5-HT_BDS,ICA_ by Student’s t-test), suggesting that Na_V_1.3 contributes to 5-HT release by EC cells. We next examined whether NaV1.3 is involved in EC cell response to luminal stimulants, such as the short chain fatty acid (SCFA) butyrate. We found that while butyrate resulted in 5-HT release (from t_0_ of 0.36 ± 0.12 ng/mL to t_20,butyrate_ of 1.21 ± 0.13 ng/mL, for Δ5-HT_butyrate_ of 0.86 ± 0.21 ng/mL, n = 3), this response did not appear to rely on Na_V_1.3 channels since ICA failed to inhibit 5-HT release (from t_0_ of 0.51 ± 0.13 ng/mL to t_20,butyrate,ICA_ of 1.32 ± 0.60 ng/mL, for a Δ5-HT_butyrate,ICA_ of 0.81 ± 0.58 ng/mL, n = 3, *p* > 0.05 between Δ5-HT_butyrate_ and Δ5-HT_butyrate,ICA_ by Student’s t-test). Our data show that while Na_V_1.3 channels contribute to 5-HT release by colonic EC cells, the mechanism upstream of Na_V_1.3 is currently undetermined.

## Discussion

The enterochromaffin (EC) cell is the single most important source of systemic serotonin (5-HT)^1^, and EC cell 5-HT plays critical physiologic roles within the GI tract and systemically^2^. However, the molecular mechanisms of EC cell function and serotonin release are poorly understood because only a few studies have examined EC cells in isolation from the rest of the GI epithelium^13,25,26^. In isolated guinea pig EC cells, there was an inward current consistent with voltage-gated calcium channels but no fast sodium current^15^, while in a different study TTX-sensitive sodium current was present in murine EC cells^13^. It is unclear whether species differences are responsible for these results.

In this study, we examined purified EC cells as a group and as single cells. We discovered that EC cells are not only electrically excitable, exhibiting spontaneous bursting electrical activity, but that their electrical excitability depends on a specific voltage-gated sodium-selective ion channel, *Scn3a*-encoded Na_V_1.3, and that Na_V_1.3 contributes to 5-HT release.

We found that *Scn3a* mRNA is a single highly expressed voltage-gated sodium channel in dissociated and FACS-sorted small bowel Tph1-CFP cells, and it was expressed in single Tph1-CFP EC cells from both small bowel and colon primary cultures. Our data also show that the Na_V_1.3 protein is present in ~90% of small bowel and colon EC cells in both human and mouse. Previous studies that examined gene expression in the GI epithelium suggested that *Scn3a* is expressed in enteroendocrine cells. *Scn3a* is expressed in intestinal neurogenin 3 (*Ngn3*)^27^ positive cells, which are epithelial cells of a secretory phenotype^28^, and chromogranin A (CgA)^13^ positive cells, which are enteroendocrine cells that include EE and EC cells^29^. More specifically, in small bowel enteroids *Tph1*+ EC cell single cell expression profiles showed that *Scn3a* was one of the most abundantly expressed ion channels^30^. The L-cell, a different type of enteroendocrine cell that produces glucagon-like peptides (GLP) and peptide YY (PYY), also expresses *Scn3a* ^18^. In contrast, *Scn3a* was not found in the enteroendocrine K cells that produce and secrete glucose-dependent insulinotropic polypeptide (GIP)^27^. Overall, our results align with a number of studies that showed *Scn3a*-encoded Na_V_1.3 is densely expressed in multiple, but not all, types of enteroendocrine cells.

Intriguingly, *Scn3a* was previously found in endocrine and neuroendocrine cells outside the enteroendocrine system, such as neuroendocrine adrenal chromaffin cells^17^ and pancreatic α- and β-cells^16^. In addition to *Scn3a* (Na_V_1.3), these endocrine cells express other Na_V_ isoforms: Na_V_1.7 for mouse α- and β-cells^16,31^, Na_V_1.6 and Na_V_1.7 for human β-cells^32^, and Na_V_1.9 for L-cells^18^. In EC cells, in addition to the highly expressed Na_V_1.3, we found only one other Na_V_ isoform, Na_V_1.6, but at much smaller expression levels. With regard to the EC cell, it is unclear if the *Scn8a*-encoded Na_V_1.6 contributes to electrophysiology. However, our data suggest that Na_V_1.3 is the most functionally significant voltage-gated sodium channel isoform, since Na_V_1.3 blockade nearly eliminated the EC cell voltage-dependent inward current and abolished action potentials.

We established and used EC cell primary cultures to determine whether EC cells from both small bowel and colon have voltage-gated inward currents. We found that as with immunohistochemistry, voltage-dependent inward currents were present in most but not all EC cells. In both small bowel and colon EC cells, Na_V_ currents were inhibited by Na^+^ substitution or by the Na_V_1.3/Na_V_1.1-selective blocker ICA-121431^23^. Since EC cells showed no Na_V_1.1 expression, we concluded that the fast voltage-dependent inward current is carried by Na_V_1.3. The biophysical properties of the inward currents were nearly identical between small bowel and colon EC cells and were similar to other endocrine cells that use Na_V_1.3 for excitability, namely adrenal chromaffin cells^17^, α- and β-cells^16^, and enteroendocrine L-cells^18^.

We were intrigued when we found that EC cells fire bursts of spontaneous action potentials. When we pursued further, we found that elicited EC cell action potentials rely on Na_V_1.3, since Na^+^ substitution and ICA-121431 eliminated EC cell excitability. Primary EC cells had a dynamic cell membrane resting potential that fluctuated between two dominant potentials. The likelihood of firing an action potential from the EC cell resting membrane potential (−72 ± 4 mV) was exceedingly low, but when the EC cell membrane potential reached a plateau (−56 ± 4 mV), a burst of action potentials quickly resulted. The bursts were self-terminating and action-potential amplitudes matched well with the voltage-dependent properties of EC cell Na_V_1.3 channels. We currently do not know the mechanism downstream of Na_V_1.3. There exist several possibilities. First, bursting could allow the EC cells to maintain a depolarized membrane potential that activates calcium channels that are critical for 5-HT release by EC cells^13–15^. While previous studies have agreed that calcium channel activation is important for 5-HT release, future studies will need to determine whether and how Na_V_1.3 activation is coupled to calcium channel activation. Second, there is a possibility of Ca^2+^-independent exocytosis that relies on cytoplasmic Na^+^ in the process of hormone secretion^33^. Third, Na_V_1.3 may be involved in cellular signaling between EC cells and extrinsic afferents or intrinsic primary afferent neurons (IPANs)^34^. Further work is required to elucidate the molecular determinants of EC cell resting and plateau potentials, the relationships between the membrane potential and intracellular calcium dynamics, and the contribution to cell-cell communication.

Given the electrical excitability of EC cells, we were interested to find out whether Na_V_1.3-mediated EC cell electrical excitability plays a role in EC cell 5-HT release. In colonic EC cells, we found that primary EC cell depolarization by KCl increased 5-HT release and KCl-induced 5-HT release was inhibited by Na_V_1.3 blocker ICA-121431. We then used an established Na_V_1.3 agonist – a sea anemone peptide toxin blood depressing substance-1 (BDS-1)^24^. BDS-1 stimulated 5-HT release from EC cells, and similar to KCl, the addition of Na_V_1.3 inhibitor ICA-121431 inhibited 5-HT release, suggesting the involvement of Na_V_1.3 channels in 5-HT release. However, 5-HT release by colonic EC cells in response to SCFA butyrate did not involve Na_V_1.3, which is consistent with the notion that SCFA chemosensation requires G-protein coupled receptors^13,35^. Our data not only fit with the existing body of literature, where GI tissues were used to examine 5-HT release, but also provide intriguing novel possibilities. Depolarization of porcine mucosa by KCl produced a steady outflow of 5-HT, and submucosally-applied tetrodotoxin (TTX) reduced 5-HT release^36^. Interestingly, TTX applied to the luminal side did not inhibit 5-HT release^37^. Similar to TTX, the Na_V_ channel activator veratridine applied to the luminal side did not affect 5-HT release^36^. Therefore, manipulation of Na_V_ channels from the luminal side by cell-impermeable substances does not inhibit EC cell 5-HT release. On the other hand, EC cell 5-HT release was effectively blocked by intraluminal application of lidocaine, a well-known lipid permeable blocker of sodium channels^37^. In our study, we found that Na_V_1.3 was always localized to the basal side and in some instances within EC cell basal extensions, recently termed “neuropods”^38^. We suspect that the divergent data on EC cell voltage-gated sodium channels affecting 5-HT release may be due to the localization of Na_V_1.3 channels at the EC cell basal surface, where hydrophilic drugs do not have access. Consistent with the idea of EC cell functional polarization, recent work shows that EC cells respond differently to luminal versus systemic glucose exposure^25^. Therefore, through localization of *Scn3a* to the basal side of EC cells, the amplification machinery of these cells is protected from luminal exposure, where there is a rich variety of potential chemical stimulants.

EC cell electrical excitability transforms the EC cell from a sensory receptacle, driven by receptor currents to activate 5-HT exocytosis, to a cell that can participate in complex bidirectional communication with the enteric and extrinsic nervous systems. In this respect, the EC cell joins the taste cells, which are also electrically excitable. In fact, *Scn3a* was also found specifically expressed at the basal side of sweet, bitter, and umami taste cells, where it is proposed to use electrical excitability to amplify currents generated by TRPM5 in response to tastant stimulation^39^, which is critical for the taste cells’ communication with afferent neurons. Interestingly, the EC cells also express taste receptors^13,40,41^ and multimodal chemosensor TRPA1^13,42^. In addition, recent evidence suggests functional connections between enteroendocrine cells with afferent neurons^13,34^. Therefore, stimulation of bursting electrical activity in EC cells may allow for a direct communication with afferent neurons. A complementary possibility is that EC cell Na_V_1.3 is required for execution of efferent neuronal control of EC cells, as vagal stimulation was previously shown to affect 5-HT release from EC cells^13,43^. Such communication between an efferent neuron and a neuroendocrine cell is exemplified by splanchnic nerve activation of chromaffin cells in the adrenal medulla, where sodium channels are critical for defining the bursting properties of chromaffin cells^17^.

Our findings that EC cell electrical excitability and bursting behavior contribute to 5-HT release not only provide a novel mechanism of EC cell function, but may also have novel therapeutic possibilities. Future studies need to examine Na_V_1.3 function in human EC cells, because if Na_V_1.3 contributes to human EC cell function, novel therapeutic possibilities would exist. For example, in diabetes, studies have established not only that manipulation of islet cell electrical excitability affects hormone secretion^32^ but that blockade of pancreatic islet sodium channels may be effective in combating diabetes^44^. An alternative therapeutic possibility is Na_V_1.3 blockade in carcinoid syndrome, as human carcinoid cells were found to highly express *SCN3A* ^45^. Since EC cell 5-HT is critical to many GI and systemic functions, blockade of EC cell Na_V_1.3 may provide novel approaches for targeting GI and systemic pathophysiologic conditions.

In summary, we show that EC cells in human and mouse densely express the *Scn3a*-encoded voltage-gated sodium channel Na_V_1.3, which is responsible for the electrical excitability and contributes to 5-HT release. The discovery of Na_V_1.3-dependent EC cell excitability may have significant potential implications for EC cell physiology, pathophysiology, and therapy.

## Methods

All experimental procedures were approved by the Institutional Review Board (IRB) and the Institutional Animal Care and Use Committee (IACUC) of the Mayo Clinic and all experiments were performed in accordance with relevant guidelines and regulations.

### Cell Culture

#### Primary EC cell culture

Tph1-CFP mice were euthanized at 5–7 weeks, and a 10-cm length of small bowel or colon was removed. Full thickness tissue was inverted, chopped, and washed three times in ice-cold PBS. The tissue was digested under agitation at 37 °C in DMEM (D6546, Sigma, St. Louis, MO), 0.1% BSA (A2153, Sigma, St. Louis, Missouri), and 0.1 mg/mL (small bowel) or 0.6 mg/mL (colon) Collagenase type XI (C9407, Sigma, St. Louis, MO) in 4 separate digestions, for a total of 30 (small bowel) or 40 (colon) minutes. Supernatants were collected from the last two digestions, spun twice at 100 rcf for 5 minutes, and suspended at 1,000,000 cells per mL in DMEM (D6546, Sigma, St. Louis, MO), 5% Heat Inactivated FBS (F4135, Sigma, St. Louis, MO), 1% Pen-step (15140122, Invitrogen, Carlsbad, CA), 1% L-Glutamine (2503008, Invitrogen, Carlsbad, CA), and 10 µM (small bowel) Y-27632 (72304, Stem Cell Technologies, Vancouver, Canada) in dishes (P35GC-1.5-14-C, MatTek Corporation, Ashland, MA) coated with 5% w/v Matrigel (354230, Corning, Corning, NY). Cells were maintained in standard culture conditions for 24–48 hours.

### Primary EC cell culture 5-HT release

Mouse colon primary EC cells cultures were grown on 12 well dishes and 5-HT release was measured in response to stimuli using enzyme linked immunoassay (ELISA, BA E-5900, Rocky Mountain Diagnostics, Colorado Springs, CO). After 24 hours of incubation, media was removed, NaCl Ringer’s solution was added and a sample was collected from each well for a baseline 5-HT reading. Fluoxetine Hydrochloride (2 µM, F132, Sigma, St. Louis, MO), a serotonin reuptake inhibitor, was preincubated in all wells for 5 minutes at 37 °C. ICA-121431 (10 µM, I-170, Alomone, Jerusalem, Israel) was also preincubated in wells that would later receive the inhibitor. Solutions were removed and 50 mM KCl (P9333, Sigma, St. Louis, MO), 1 µM BDS-1 (B-400, Alomone, Jerusalem, Israel) and 5 mM Butyrate (B5887, Sigma, St. Louis, MO) with and without 10 µM ICA-121431 were added to the wells and incubated for 20 minutes at 37 °C. Samples were then collected from all wells and 1% 5-HT stabilizer (BA E-5937, Rocky Mountain Diagnostics, Colorado Springs, CO) was added to each sample. Samples were spun for 5 minutes at 5000 rpm and stored at −80 °C. 5-HT release was measured by a serotonin enzyme immunoassay (BA E-5900, Rocky Mountain Diagnostics, Colorado Springs, CO) according to manufacturer’s instructions. Absorbance was measured at 450 nm and concentrations were determined using a standard curve.

#### Intestinal epithelial single cell isolation and cell sorting

Mouse intestinal epithelial cells were isolated as previously described with modification^22^. The intestinal samples (~10 cm long pieces of duodenum/jejunum) were collected from Tph1-CFP BAC transgenic mice and cut into 2-cm small pieces. The mucosa suspensions were obtained by incubating the intestinal pieces in dissociation buffer (30 mM EDTA, 1.5 mM DTT in PBS) at room temperature for 30 minutes. Single cell suspension was generated from intestinal mucosa by digestion in protease buffer containing 50 µg pronase (Sigma-Aldrich) per 100 mL Basal Medium Eagle (Gibco). Cells were sorted by flow cytometry using a BD FACSAria II cell sorter. CFP-positive single cells were collected by gating for CFP fluorescence combined with side and forward scatter to select single cells. Non-virable cells were excluded by gating for 7-amino actinomycin D (7-AAD, BD Biosciences) shortly after treatment with 7-AAD for 5 minutes before analysis.

### Molecular Biology

#### Sorted TPH1-CFP qPCR

We employed standard qPCR techniques on sorted EC cells. Total RNA from FACS sorted Tph1-CFP cells was extracted using Qiagen RNeasy Micro kit (Valencia, CA, USA) according to the manufacturer’s protocol. cDNA was reversed transcribed using Maxima cDNA Synthesis kit (Thermofisher). qPCR reaction was prepared with iTaq Universal SYBR green supermix (BioRad) and amplified for 40 cycles using CFX96 Real Time System (BioRad). Any sample that did not generate melting temperature was considered as no target gene expression, and its Ct value was assigned to 40. Target gene expression levels were normalized to that of reference gene *Actb*, resulting in a Ct difference (ΔCt) that was used to determine relative expression level of each target gene. Primers for the sodium channel α-subunits and Tph1 are listed in Supplementary Table 1.

#### Single cell RT-qPCR

Single cell RT-qPCR was performed with an Ambion Single Cell-to-C_t_ kit (4458236, ThermoFisher, Grand Island, NY). *Scn3a* and *Tph1* gene expression was analyzed in mixed enterochromaffin cell primary cultures using single cell RT-qPCR. Media was removed from cultures after 24 h and replaced with NaCl Ringer solution. Individual cells were aspirated into a glass pipette by negative pressure from a syringe, and then ejected by positive pressure into a PCR tube, subsequently placed on dry ice. Cells were collected to a total of 2–6 CFP^+^ cells, 2–6 CFP^−^ cells, or 0 cells (buffer medium only) per tube. First, samples were lysed using Single Cell DNase I/Single Cell Lysis Solution. Then, cDNA was synthesized using Single Cell SuperScript RT and a thermal cycler protocol that consisted of 10 min at 25 °C, 60 min at 42 °C, and 5 min at 85 °C. Next, a pre-amplification step using a 0.2x mixture of *Scn3a* and *Tph1* primers (Supplementary Table 1) was added to each sample and run for 10 min at 95 °C for enzyme activation, then 14 cycles of 15 s at 95 °C and 4 min at 55 °C for amplification, and finally 10 min at 99 °C for enzyme deactivation. After pre-amplification products were diluted 1:10, a RT-qPCR reaction was run on a LightCycler 480 (Roche, Indianapolis, Indiana) using 480 sybr green (Roche 04707516001, Indianapolis, IN) and *Scn3a* and *Tph1* primers for 2 min at 50 °C, 10 min at 95 °C, and 40 cycles of 5 s at 95 °C and 1 min at 55 °C. Finally, PCR products were separated by 2% agarose gel electrophoresis. All bands were sequenced to confirm identity.

### Immunohistochemistry

#### Tissue labeling

Tph1-CFP mouse tissues were cut into 1 cm × 0.5 cm flat sheets or 0.5 cm length tubes from colon and small bowel. Human colon and small bowel tissue samples were collected from surgical waste. All tissues were fixed in 4% paraformaldehyde phosphate buffer (PFA) for 4 hours, rinsed in phosphate buffered saline (PBS), incubated overnight in 30% sucrose in PBS at 4 °C, frozen in 2-methylbutane over dry ice in optimum cutting temperature (OCT) embedding compound (Sakura Finetek, Torrance, CA), and stored at −80 °C until sectioned. Blocks were then sectioned into 12 µm thickness sections and thawed onto slides. Human tissue slides underwent antigen retrieval using target retrieval solution, as per manufacturer’s protocol (Dako, Carpinteria, CA), and background reduction process with 3% hydrogen peroxide for 5 min. All slides of mouse and human tissue were then rinsed with PBS twice for 5 min and blocked with 200 µL/slide of 1% bovine serum albumin (BSA)/PBS/0.3% Triton in a humidity chamber for 1 hour. Primary antibodies (Supplementary Table 2) were added at 200 µL/slide of BSA/PBS/0.3% Triton and were incubated at 4 °C overnight in a humidity chamber. Slides were then rinsed 5 times for 3 min with PBS. Secondary antibodies (Supplementary Table 2) at 200 µL/slide of BSA/PBS/0.3% Triton were incubated for 1 h in the dark. Slides were again rinsed 5 times for 3 min with PBS and mounted in slowfade gold with 4’,6-diamidino-2-phenylindole (DAPI, Life Technologies, Grand Island, NY) mounting buffer. Control slides were also prepared, on which no primary antibody was used. Primary and secondardy antibodies are listed in Supplementary Tables 1 and 2, respectively.

#### Cell counting

To quantify Na_V_1.3 localization in EC cells, Tph1-CFP and/or Na_V_1.3 positive cells in mouse tissue and 5-HT and/or Na_V_1.3 positive cells in human tissue were counted in epithelium only, as defined by the DAPI positive outer layer of the mucosa facing the lumen. Images were taken on Olympus BX51W1 epifluorescent (40x) and Olympus FV1000 confocal (20x, 0.95 NA and 60x, 1.2 NA, z-res 0.91 µm) microscopes (Olympus Corporation, Tokyo, Japan). We counted EC cells from the small bowel and colon of 3 mice and 3 humans.

### Electrophysiology

#### Data acquisition

Electrodes (Kimble KG12 glass) were pulled with a Sutter P97 puller (Sutter Instruments, Novato, CA), coated with R6101 (Dow Corning, Midland, MI), and fire polished to 2–5 MΩ. Electrophysiology data were acquired with an Axopatch 200B patch clamp amplifier, CyperAmp 320 signal conditioner, Digidata 1322A digitizer, and pClamp 10.6 software (Molecular Devices, Sunnyvale, CA).

#### Solutions

NaCl Ringer’s solution contained (in mM): 150 Na^+^, 160 Cl^−^, 5 K^+^, 2.5 Ca^2+^, 10 HEPES, 5.5 glucose, pH 7.35 (with NaOH) and 310 mmol/kg. For whole cell electrophysiology, the extracellular solution contained (in mM): 100 Na^+^, 10 NMDG^+^, 5 Cs^+^, 5 K^+^, 10 Ca^2+^, 5 Mg^2+^, 0.01 Gd^3+^, 150 Cl^−^, 15 HEPES, and 5.5 glucose, pH 7.35 (with HCl), 270 mmol/kg. The intracellular solution contained (in mM): 70 Cs^+^, 50 K^+^, 5 Na^+^, 10 Mg^2+^, 1.2 Ca^2+^, 120 CH_3_SO_3_^−^, 30 Cl^−^, 15 HEPES, and 5 EGTA to buffer [Ca^2+^]_i_ to 300 nM, pH 7.0 with CsOH, 290 mmol/kg. With these solutions, the predicted reversal potential for Na^+^ was + 76 mV. The predicted liquid junction potential was subtracted during analysis.

#### Voltage-clamp protocols

The signals were filtered at 4 kHz and sampled at 20 kHz. Data were recorded from cells held at −120 mV, stepped for 50 ms to −80 through +15 mV in 5 mV intervals to measure steady state activation, then stepped to 0 mV for 50 ms to measure steady state inactivation. The start-to-start time was 250 ms per sweep and 6 s per run for up to 10 runs.

#### Current-clamp protocols

To examine elicited activity, EC cells were injected with −10 pA current to hyperpolarize the baseline membrane potential to −60 to −80 mV, then action potentials were elicited every second by 50-ms injections of depolarizing current in 2 pA intervals. To examine spontaneous activity, cells were injected with zero current, and the membrane potential was recorded in gap-free mode.

#### Data analysis

Data were analyzed with pClamp 10.6 (Molecular Devices), Microsoft Excel 2010 and SigmaPlot 12 (Systat Software, San Jose, CA). Peak currents are expressed relative to cell capacitance (pA/pF). Current-voltage (IV) data were fit by the Boltzmann function, *I*(*V*) = (*V* *−* *V*_*REV*_)*/*(*1* + *exp*((*V* − *V*_*1/*2*a*_)*/δV*_*A*_))), where *I* is the peak current (pA/pF) at the test voltage *V* (mV), *V*_*REV*_ is the reversal potential, *V*_*1/2A*_ is the half-activation voltage, and *δV*_*A*_ is the voltage sensitivity of activation (the “slope”). For kinetic analysis, the currents (*I* in pA) were fit at 0 mV to the equation *I*(*t*) = *A*_*1*_ *exp*(*−t/τ*_*A*_) + *A*_*2*_ *exp*(*−t/τ*_*F*_) + *A*_3_ *exp*(*−t/τ*_*S*_) + *C*, where *τ*_*A*_ was the activation constant; *τ*_*F*_ and *τ*_*S*_, fast and slow inactivation time constants (ms); and *A*_*X*_ and *C*, constants. All-point histograms of spontaneous membrane potential activity were fit with a 2-parameter Gaussian function, f(x) = A_1_(e^−(x − µ1)(x − µ1)/(2(σ1)(σ1))^)/(σ_1_(2π)^0.5^) + A_2_(e^−(x − µ2)(x − µ2)/(2(σ2)(σ2))^)/(σ_2_(2π)^0.5^) + C, where µ_1_ and µ_2_ are the resting and plateau potentials, respectively.

## Electronic supplementary material

Supplementary Information

## References

[1] Cote F (2003). Disruption of the nonneuronal tph1 gene demonstrates the importance of peripheral serotonin in cardiac function. Proc. Natl. Acad. Sci. USA.

[2] Mawe GM, Hoffman JM (2013). Serotonin signalling in the gut–functions, dysfunctions and therapeutic targets. Nature Reviews: Gastroenterology and Hepatology.

[3] Heredia DJ (2013). Important role of mucosal serotonin in colonic propulsion and peristaltic reflexes: *in vitro* analyses in mice lacking tryptophan hydroxylase 1. J. Physiol.

[4] Heredia DJ, Dickson EJ, Bayguinov PO, Hennig GW, Smith TK (2009). Localized release of serotonin (5-hydroxytryptamine) by a fecal pellet regulates migrating motor complexes in murine colon. Gastroenterology.

[5] Keating DJ, Spencer NJ (2010). Release of 5-hydroxytryptamine from the mucosa is not required for the generation or propagation of colonic migrating motor complexes. Gastroenterology.

[6] Spencer NJ (2011). Mechanisms underlying distension-evoked peristalsis in guinea pig distal colon: is there a role for enterochromaffin cells?. American journal of physiology. Gastrointestinal and liver physiology.

[7] Launay JM (2002). Function of the serotonin 5-hydroxytryptamine 2B receptor in pulmonary hypertension. Nat. Med..

[8] Yadav VK (2010). Pharmacological inhibition of gut-derived serotonin synthesis is a potential bone anabolic treatment for osteoporosis. Nat. Med..

[9] Crane JD (2015). Inhibiting peripheral serotonin synthesis reduces obesity and metabolic dysfunction by promoting brown adipose tissue thermogenesis. Nat. Med..

[10] Sumara G, Sumara O, Kim JK, Karsenty G (2012). Gut-derived serotonin is a multifunctional determinant to fasting adaptation. Cell Metab..

[11] Margolis, K. G. *et al*. Pharmacological reduction of mucosal but not neuronal serotonin opposes inflammation in mouse intestine. *Gut* (2013). 10.1136/gutjnl-2013-304901 PMC4034681 23749550

[12] Racke K, Reimann A, Schworer H, Kilbinger H (1996). Regulation of 5-HT release from enterochromaffin cells. Behav. Brain Res..

[13] Bellono NW (2017). Enterochromaffin Cells Are Gut Chemosensors that Couple to Sensory Neural Pathways. Cell.

[14] Lomax RB, Gallego S, Novalbos J, Garcia AG, Warhurst G (1999). L-Type calcium channels in enterochromaffin cells from guinea pig and human duodenal crypts: an *in situ* study. Gastroenterology.

[15] Raghupathi R (2013). Identification of unique release kinetics of serotonin from guinea-pig and human enterochromaffin cells. J. Physiol.

[16] Zhang Q (2014). Na+ current properties in islet alpha- and beta-cells reflect cell-specific Scn3a and Scn9a expression. J. Physiol.

[17] Vandael DH (2015). Reduced availability of voltage-gated sodium channels by depolarization or blockade by tetrodotoxin boosts burst firing and catecholamine release in mouse chromaffin cells. J. Physiol.

[18] Rogers GJ (2011). Electrical activity-triggered glucagon-like peptide-1 secretion from primary murine L-cells. J. Physiol.

[19] Strege, P. R. *et al*. Mouse Colon Enterochromaffin (EC) Cells Express Voltage-Gated Sodium Channels and Are Electrically Excitable. *Gastroenterology* **150**, 10.1016/S0016-5085(16)30282-7 (2016).

[20] Strege PR (2017). SCN3A-encoded voltage-gated sodium channel NaV1.3 bestows mouse enterochromaffin cells with patterns of bursting electrical activity. Gastroenterology.

[21] Strege PR (2017). SCN3A-encoded Voltage-gated Sodium Channel Na(V)1.3 is Specifically Expressed in Human and Mouse Gastrointestinal Enterochromaffin Cells and is Important for Enterochromaffin Cell Excitability. FASEB J..

[22] Li HJ (2014). Distinct cellular origins for serotonin-expressing and enterochromaffin-like cells in the gastric corpus. Gastroenterology.

[23] McCormack K (2013). Voltage sensor interaction site for selective small molecule inhibitors of voltage-gated sodium channels. Proc. Natl. Acad. Sci. USA.

[24] Liu P, Jo S, Bean BP (2012). Modulation of neuronal sodium channels by the sea anemone peptide BDS-I. J. Neurophysiol..

[25] Zelkas L (2015). Serotonin-secreting enteroendocrine cells respond via diverse mechanisms to acute and chronic changes in glucose availability. Nutr. Metab. (Lond.).

[26] Kidd M, Modlin IM, Eick GN, Champaneria MC (2006). Isolation, functional characterization, and transcriptome of Mastomys ileal enterochromaffin cells. Am. J. Physiol. Gastrointest. Liver Physiol..

[27] Sommer CA, Mostoslavsky G (2014). RNA-Seq analysis of enteroendocrine cells reveals a role for FABP5 in the control of GIP secretion. Mol. Endocrinol..

[28] Li HJ, Ray SK, Singh NK, Johnston B, Leiter AB (2011). Basic helix-loop-helix transcription factors and enteroendocrine cell differentiation. Diabetes Obes. Metab..

[29] Fothergill, L. J., Callaghan, B., Hunne, B., Bravo, D. M. & Furness, J. B. Co-storage of enteroendocrine hormones evaluated at the cell and subcellular levels in male mice. *Endocrinology*, 10.1210/en.2017-00243 (2017). 10.1210/en.2017-00243 28430903

[30] Grun D (2015). Single-cell messenger RNA sequencing reveals rare intestinal cell types. Nature.

[31] Vignali S, Leiss V, Karl R, Hofmann F, Welling A (2006). Characterization of voltage-dependent sodium and calcium channels in mouse pancreatic A- and B-cells. J. Physiol.

[32] Braun M (2008). Voltage-gated ion channels in human pancreatic beta-cells: electrophysiological characterization and role in insulin secretion. Diabetes.

[33] Nordmann JJ, Stuenkel EL (1991). Ca(2+)-independent regulation of neurosecretion by intracellular Na+. FEBS Lett..

[34] Bohorquez DV (2015). Neuroepithelial circuit formed by innervation of sensory enteroendocrine cells. J. Clin. Invest..

[35] Karaki S (2006). Short-chain fatty acid receptor, GPR43, is expressed by enteroendocrine cells and mucosal mast cells in rat intestine. Cell Tissue Res..

[36] Racke K, Schworer H (1993). Characterization of the role of calcium and sodium channels in the stimulus secretion coupling of 5-hydroxytryptamine release from porcine enterochromaffin cells. Naunyn-Schmiedeberg’s archives of pharmacology.

[37] Fukumoto S (2003). Short-chain fatty acids stimulate colonic transit via intraluminal 5-HT release in rats. American journal of physiology. Regulatory, integrative and comparative physiology.

[38] Bohorquez DV (2014). An enteroendocrine cell-enteric glia connection revealed by 3D electron microscopy. PLoS One.

[39] Gao N (2009). Voltage-gated sodium channels in taste bud cells. BMC Neurosci..

[40] Schafermayer, A., Zanner, R., Graztl, M., Sachs, G. & Prinz, C. In *Cell Biology of the Chromaffin Cell* (eds Borges, R. & Gandia, L.) 175–186 (Instituto Teófilo Hernando, 2004).

[41] Sutherland K, Young RL, Cooper NJ, Horowitz M, Blackshaw LA (2007). Phenotypic characterization of taste cells of the mouse small intestine. Am. J. Physiol. Gastrointest. Liver Physiol..

[42] Nozawa K (2009). TRPA1 regulates gastrointestinal motility through serotonin release from enterochromaffin cells. Proc. Natl. Acad. Sci. USA.

[43] Pettersson G (1979). The neural control of the serotonin content in mammalian enterochromaffin cells. Acta Physiol. Scand. Suppl..

[44] Dhalla, A. K. *et al*. Blockade of Na+ Channels in Pancreatic alpha-Cells has Anti-Diabetic Effects. *Diabetes*, 10.2337/db13-1562 (2014). 10.2337/db13-1562 24812428

[45] Kidd M, Modlin IM, Drozdov I (2014). Gene network-based analysis identifies two potential subtypes of small intestinal neuroendocrine tumors. BMC Genomics.

