# Supplementary material for: Sodium channel NaV1.3 is important for enterochromaffin cell excitability and serotonin release
Source: Sci Rep. 2017 Nov 15;7:15650. doi: 10.1038/s41598-017-15834-3 (PMC5688111; doi:10.1038/s41598-017-15834-3)
Supplement: Supplementary file 1 — Supplementary Information [file 41598_2017_15834_MOESM1_ESM.pdf]

SUPPLEMENTARY INFORMATION

Sodium channel $Na_V1.3$ is important for enterochromaffin cell excitability and serotonin release

Peter R. Strege,[1*] Kaitlyn Knutson,[1*] Samuel J. Eggers,[1] Joyce H. Li,[2] Fan Wang,[1,3] David Linden,[1] Joseph H. Szurszewski,[1] Lorin Milescu,[4] Andrew B. Leiter,[2] Gianrico Farrugia,[1] Arthur Beyder[1#]

[1]Enteric Neuroscience Program, Division of Gastroenterology & Hepatology, Department of Physiology & Biomedical Engineering, Mayo Clinic, Rochester, Minnesota

[2]Division of Gastroenterology, Department of Medicine, University of Massachusetts Medical School, Worcester, Massachusetts

[3]Department of Gastroenterology, Shanghai Tenth People's Hospital, Tongji University School of Medicine, 300 Yanchang Middle Road, Shanghai, P.R. China. 200072;

[4]Division of Biological Sciences, University of Missouri, Columbia, Missouri

*Authors contributed equally to this work

Short title: The excitable EC cell

Corresponding author:

Arthur Beyder, M.D., Ph.D.[#]
Enteric NeuroScience Program
Division of Gastroenterology & Hepatology
Departments of Medicine and Physiology and Biomedical Engineering
Mayo Clinic
200 First Street SW
Rochester, MN 55905, USA.
Telephone: (507) 284-2511, Fax: (507) 284-0266
Email: beyder.arthur@mayo.edu

[#]lead author

Primer Sequence (5'-3')

	Forward	Reverse
**Scn1a**	CATTTTTGTCCCTGTTTCGACTG	TGCCAGCAGCACGC
**Scn2a**	ATCTGCCTCAACATGGTGAC	GGTGAACAGGACGATGAACA
**Scn3a**	TCCGAGCCTTATCCCGCTTTGA	GAAGATGAGGCACACCAGTAGC
**Scn4a**	GAAAACCATCACGGTCATCC	TCCGAGAGCTTTTTCACAGAC
**Scn5a**	GCCAGATCTCTATGGCAACC	TTGCCCTTATTCAGCACGAT
**Scn8a**	ATGGGGTAGGCTCTCCGAG	CCGACTCTGACTTAAACACCTTC
**Scn9a**	GCTGAGCCTATCAATGCAGA	ACTTGGCAGCATGGAAATCT
**Scn10a**	TGGGTAGCTTATGGCTTCAAA	CTATGAGGCTTGTGAGGGAGA
**Scn11a**	TGGCCCTAACGCTTTTTCCA	CCATCGGGGCAGGATCTTC
**Tph1**	TGTTGACTGCGACATCAGCCGA	GGAAACCAAGGGACAGTCTCCA

Supplementary Table 1. PCR primers used in this study.

Human Colon

	Source	Titer	Secondary	Source	Titer
**Na_V1.3**	Alomone (ACS-004)	Colon: 1:100 (10 µg/mL) Small bowel: 1:400 (2.5 µg/mL)	Cy3	Jackson ImmunoReseach (111-165-003)	1:800 (1.25 µg/mL)
**5-HT**	Abcam (ab66047)	1:2000 (0.25 µg/mL)	Cy5	Jackson ImmunoResearch (705-175-147)	1:800 (1.5 µg/mL)

Mouse Colon and Small bowel

	Source	Titer	Secondary	Source	Titer
**Na_V1.3**	Alomone (ACS-004)	1:100 (10 µg/mL)	Cy3	Jackson ImmunoReseach (111-165-003)	1:800 (1.25 µg/mL)

Supplementary Table 2. Antibodies used in this study.

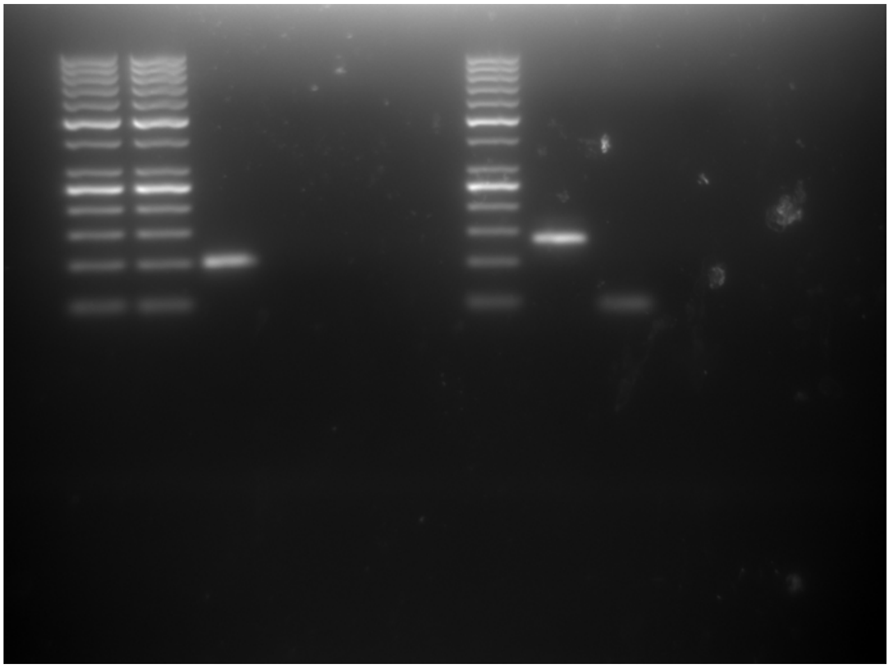

Supplementary Figure 1. Full size RT-PCR gel for Figure 4A.

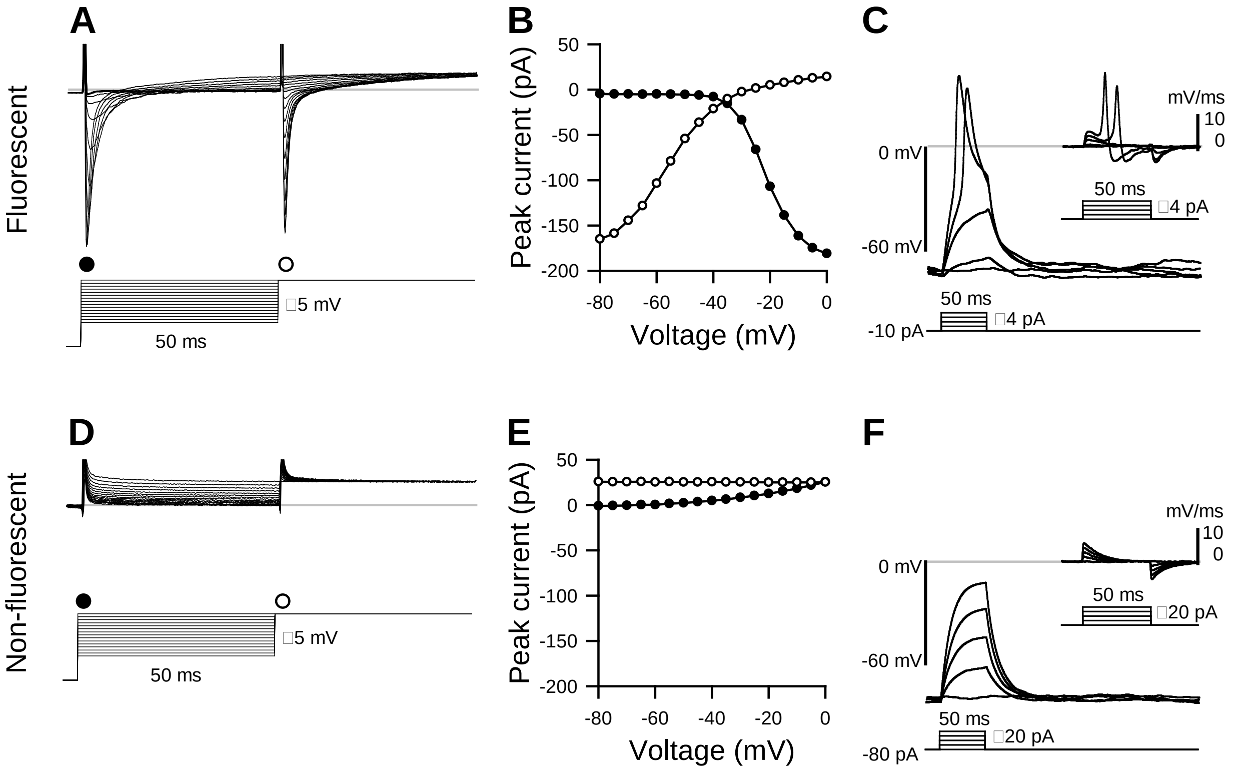

Supplementary Figure 2. Voltage-dependent inward currents and action potentials present in CFP+ EC cells but not in CFP$^-$ cells. (A) Two-pulse protocol showing the voltage-dependence of activation (filled circle) and voltage-dependence of inactivation (open circle) in a CFP+ EC cell. (B) Current-voltage (IV) relationship for voltage-dependence of activation (filled circles) and voltage-dependence of inactivation (open circles) for the same CFP+ EC cell as in (A). (C) In the same CFP+ EC cell, evoked action potentials (inset is dV/dt). (D-F) CFP$^-$ cell in the same preparation as cell in (A-C), showing a lack of voltage-dependent currents (D), the resulting horizontal IV curves (E), and a lack of action potentials elicited by current stimuli (F).

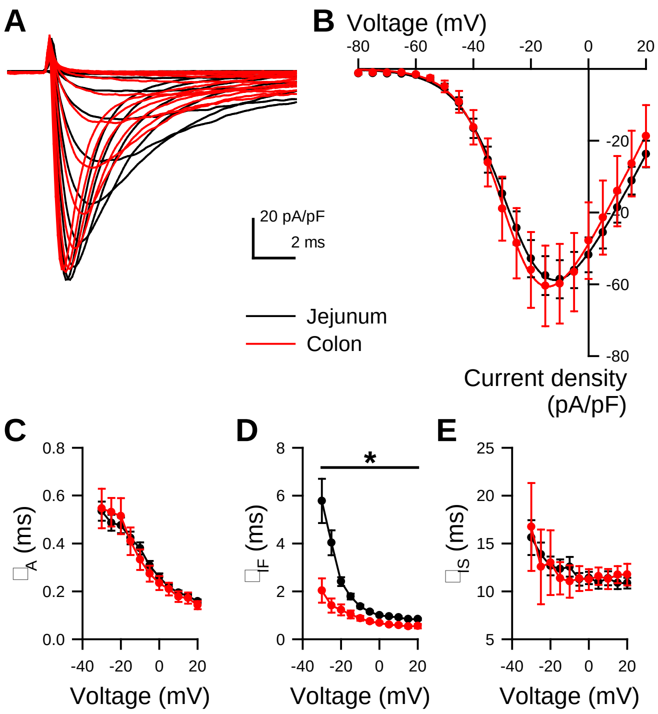

Supplementary Figure 3. Colon and small bowel EC cell voltage-dependent currents are similar except for inactivation kinetics. (A) Set of typical voltage-dependent colon (red) and small bowel (black) inward currents. (B) Current-voltage (IV) relationships for colonic and small bowel EC cells are identical. (C) Time constants of activation (τ_A) for colon and small bowel voltage-dependent currents were identical. (D) The fast constants of inactivation (τ_F) for colon EC cell inward current were faster than small bowel. (E) The slow constants of inactivation (τ_S) for colon EC cell inward current was identical were identical.

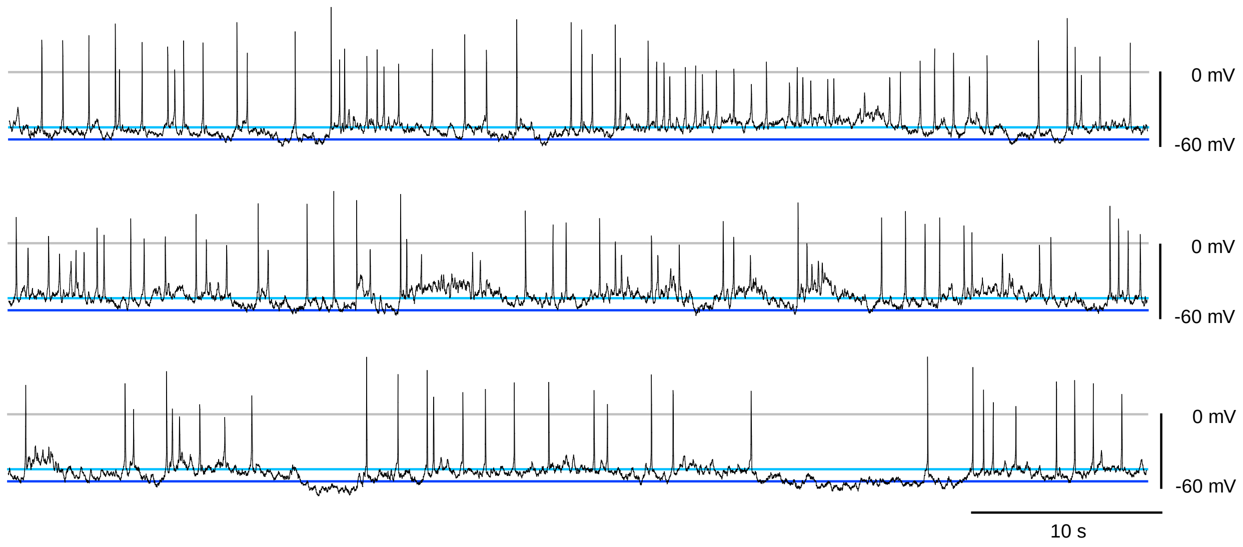

Supplementary Figure 4. Bursting action potentials from a single CFP+ EC cell lasting ~3 minutes. A dynamic cell membrane resting potential fluctuated between two dominant potentials, resting (*blue*, -55 mV) and plateau (*cyan*, -45 mV), from which action potentials fired (*gray*, 0 mV).

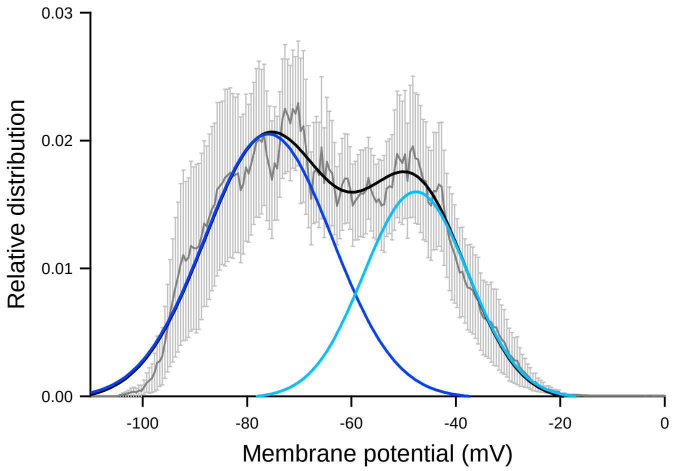

Supplementary Figure 5. All-points histogram from 11 CFP+ EC cells. Blue line is Gauss fit of resting membrane potential (Vm = -72±4 mV). Cyan line is Gauss fit of plateau membrane potential (Vm = -56±4 mV). Black line is combination of both fits.
